# Costs and effects of two public sector delivery channels for long-lasting insecticidal nets in Uganda

**DOI:** 10.1186/1475-2875-9-102

**Published:** 2010-04-20

**Authors:** Jan H Kolaczinski, Kate Kolaczinski, Daniel Kyabayinze, Daniel Strachan, Matilda Temperley, Nayantara Wijayanandana, Albert Kilian

**Affiliations:** 1 Malaria Consortium - Africa Regional Office, PO Box 8045, Plot 2, Sturrock Road, Kampala, Uganda; 2 Disease Control and Vector Biology Unit, London School of Hygiene & Tropical Medicine, Keppel Street, London, UK

## Abstract

**Background:**

In Uganda, long-lasting insecticidal nets (LLIN) have been predominantly delivered through two public sector channels: targeted campaigns or routine antenatal care (ANC) services. Their combination in a mixed-model strategy is being advocated to quickly increase LLIN coverage and maintain it over time, but there is little evidence on the efficiency of each system. This study evaluated the two delivery channels regarding LLIN retention and use, and estimated the associated costs, to contribute towards the evidence-base on LLIN delivery channels in Uganda.

**Methods:**

Household surveys were conducted 5-7 months after LLIN distribution, combining questionnaires with visual verification of LLIN presence. Focus groups and interviews were conducted to further investigate determinants of LLIN retention and use. Campaign distribution was evaluated in Jinja and Adjumani while ANC distribution was evaluated only in the latter district. Costs were calculated from the provider perspective through retrospective analysis of expenditure data, and effects were estimated as cost per LLIN delivered and cost per treated-net-year (TNY). These effects were calculated for the total number of LLINs delivered and for those retained and used.

**Results:**

After 5-7 months, over 90% of LLINs were still owned by recipients, and between 74% (Jinja) and 99% (ANC Adjumani) were being used. Costing results showed that delivery was cheapest for the campaign in Jinja and highest for the ANC channel, with economic delivery cost per net retained and used of USD 1.10 and USD 2.31, respectively. Financial delivery costs for the two channels were similar in the same location, USD 1.04 for campaign or USD 1.07 for ANC delivery in Adjumani, but differed between locations (USD 0.67 for campaign delivery in Jinja). Economic cost for ANC distribution were considerably higher (USD 2.27) compared to campaign costs (USD 1.23) in Adjumani.

**Conclusions:**

Targeted campaigns and routine ANC services can both achieve high LLIN retention and use among the target population. The comparatively higher economic cost of delivery through ANC facilities was at least partially due to the relatively short time this system had been in existence. Further studies comparing the cost of well-established ANC delivery with LLIN campaigns and other delivery channels are thus encouraged.

## Background

Regular use of long-lasting insecticidal nets (LLINs) is one of the most effective ways of preventing malaria infection [1]. This and other evidence has resulted in a considerable increase in funding for malaria control, and for LLINs in particular [2]. These new resources provide an important means towards the Roll Back Malaria target for 2010 of protecting 80% of the at-risk population with locally appropriate vector control methods [3]. In 2000, most countries in sub-Saharan Africa had very low coverage of insecticide-treated nets (ITN), but with the new resources it was feasible to considerably increase coverage, particularly over the past years [4], largely through campaign distribution of nets provided free-of-charge [5].

The challenge now faced by programme managers across sub-Saharan Africa is to continue to increase LLIN coverage and use so that all inhabitants of malaria endemic areas are protected, while also ensuring that high coverage can be sustained over time [6]. In an attempt to address these two priorities, some countries, including Uganda, have now adopted a "mixed-model" approach. Under this model, campaigns are used to rapidly increase coverage, while routine delivery (for example to target groups through health facilities) and the commercial sector are expected to maintain coverage [7]. Although this approach is intuitively appealing, there is limited evidence on the (cost-) effectiveness of different delivery channels and how they compare in different settings [8]. Where different delivery sectors (public, mixed public private, private and community-based) and delivery channels (routine service, campaigns, vouchers, etc.) have been compared, it was reported that integration into vaccination campaigns seemed the most efficient way to increase coverage, while other approaches were as or more cost-effective and seemed better suited to sustaining coverage [9].

Coverage can be defined in more than one way: i) the number of nets delivered to a population of known size; ii) ownership of LLIN by such populations, or iii) the proportion of the population that regularly use these nets. Ultimately it is regular use that reduces the risk of malaria infection and determines impact on the malaria burden, although the relationship between the level of use and protection obtained is unknown. A critical indicator to measure in the evaluation of LLIN distribution programmes is therefore the proportion of delivered nets that are actually in regular use [10,11]. This is particularly important because it has been shown that the majority of the target group generally retains the net [12-16], but that this ownership does not automatically translate into regular use [10,15-19].

To contribute to the evidence-base on LLIN delivery systems in Uganda, the present study was designed to measure key outcome indicators for distribution through stand-alone campaigns and routine antenatal care (ANC) services. The study set out to: i) determine the level of LLIN retention and use achieved by delivering nets via the two channels, ii) establish the major determinants affecting LLIN retention and use, iii) calculate the total cost of each distribution, and iv) investigate variations in costs between the two campaigns. These data were then used to calculate the cost of delivering LLINs to the population as a whole, or to the specific target groups.

## Methods

### Study areas

The study was conducted in two malaria endemic districts of Uganda, Adjumani and Jinja. Adjumani, in north-western Uganda, was included because it was one of the first to benefit from LLIN distribution campaigns in Uganda, with funds from the Global Fund to Fight AIDS, Tuberculosis and Malaria (GFATM), and because distribution of LLINs through ANC clinics has been ongoing since 2006. Jinja district, located just east of Kampala, was included because experience gained with campaign distribution in Adjumani and elsewhere was used to improve programme implementation, most notably the materials and methods for Information, Education and Communication (IEC) and Behaviour Change Communication (BCC). Jinja is also much closer to Kampala than Adjumani, allowing comparison of costs between different settings.

### LLIN distributions

Data on two different delivery channels, and three distinct distributions, were examined: two stand alone campaigns and one on-going routine delivery through ANC clinics over a 12-month period (Table 1). All distributions were supported by Malaria Consortium and conducted in partnership with the Ugandan Ministry of Health's (MoH) National Malaria Control Programme (NMCP) and District Health Teams (DHT).

**Table 1:** Overview of the three LLIN distributions studied

	Routine ANC	GFATM funded campaign	MNM funded campaign
District	Adjumani	Adjumani	Jinja
Administrative area covered^1^	All 29 district ANC facilities	2 of the 6 sub-counties in the district	1 of the 11 sub-counties in the district
Distribution method	On-going distribution to pregnant women attending their 1^st ^ANC visit	One-off campaign with house-to-house registration followed by distribution from fixed parish distribution points over two days	One-off campaign with house-to-house registration followed by distribution from fixed parish distribution points over two days
Target groups	Pregnant women	Households with pregnant women or children under five	Households with pregnant women or children under five
Dates of distribution	On-going since Jan 2007. LLINs delivered from Jan to Dec 2007 considered for costing; LLINs delivered during Feb - Apr 2007 followed-up for retention and use	March 2007	September 2007
Number of LLINs delivered	15,188^2^	16,378	12,994
Type of LLIN	PermaNet^® ^2.0	Olyset^®^	PermaNet^® ^2.0
Donor	USAID	GFATM	Malaria No More (MNM) through USAID

^1^MoH policy in Uganda is to conduct campaign LLIN distribution by sub-county across the country. Initially campaigns were designed to include as many districts as possible and therefore only a proportion of sub-counties in each districts were covered. As more LLINs became earmarked for campaign distribution the policy shifted to "filling-in" sub-counties to ensure that as many districts as possible have benefitted from campaigns in all their sub-counties.

^2 ^At the time of writing this distribution mechanism is on-going in Adjumani district. For the purposes of the study only the first year of activities was considered; the number of LLINs delivered was the number delivered over the first year.

The LLIN products were Olyset^® ^(Sumitomo Chemical Co., Japan), distributed by campaign in Adjumani, and PermaNet^® ^2.0 (Vestergaard-Frandsen, Denmark) distributed through ANC facilities in Adjumani and campaign in Jinja. Both products were removed from the manufacturers' packaging just before distribution in an attempt to promote immediate use and to minimize resale (Table 2).

**Table 2:** Details of the two campaign distributions studied

Component	GFATM funded campaign	MNM funded campaign
Procurement	Managed by WHO	Managed by Malaria Consortium
Transport to district	Sub-contracted to transport company	Sub-contracted to transport company
Storage	District MoH stores; LLINs delivered to distribution point on day of distribution	
Training of trainers	Two day meeting in Kampala	One day meeting in Kampala
District sensitization and training	20 district leaders sensitized through half day sensitization; two trainers per sub-county attended full day for sensitization and training	
Sub-county sensitization and training	Two sub-county trainers led the meeting, supervised by Malaria Consortium and central trainers. Parish and village leaders attended half-day sensitization; CMDs attended full day for sensitization and training.	
Registration	Over two days, two community medicine distributors (CMD) per village visited each household and completed a registration form detailing size of household and details of household members, noting whether these fell into the target groups of pregnant women (PW) and children under five (U5). Sub-county supervisors, central trainers and Malaria Consortium staff supervised the activity.	
Allocation	Registration lists were reviewed at one-day parish meetings. A pre-assigned number of LLINs was made available to each parish. Pre-defined allocation rules were followed at this meeting to determine how these nets would be allocated within the parish based on number of target groups in each household. The three-step allocation rules stated: (1) every household with a PW or U5 to be allocated at least one net; (2) a second LLIN to be allocated to each household with more than one target group; (3) a third LLIN to be allocated to each household with one PW and at least two U5. No more than three LLINs to be assigned to each household. If there are insufficient LLIN to complete the full three-step allocation then age of beneficiaries is used to prioritize which household are allocated under steps 2 and 3.	
Distribution	Distribution points were located at parish level. CMDs from each village presented their allocation lists. Community members arriving to receive LLINs dealt with the CMD from their own village, their name was checked off the allocation list and a signature given. The LLINs was provided without the manufacturers packaging. Distribution took place during one day.	
Post distribution follow up	Not conducted	Two CMDs per village were asked to visit around 50% of beneficiary households giving advice on use and hanging. Limited funds were available for this component, supervision was minimal and it was not possible to establish to what extent this activity took place.
IEC approach	• Sensitization of district, parish and village leaders	• Sensitization of district, parish and village leadership
	• Health educators and practical demonstrations at distribution points	• Health educators and practical demonstrations at distribution points
	• Print materials designed by MoH and provided in English, including:	• Print materials designed and pre-tested for use in other Malaria Consortium activities were used, these included:
	- Posters at distribution points	- Posters at distribution points
	- Leaflets on LLIN benefits and use distributed by community leaders	- Leaflets on net benefits and use distributed by community leaders
	- Handout with three key messages on net use, provided with each LLIN	- Handout with three key messages on net use, provided with each net

#### Campaigns

During 2007, Uganda's MoH began implementing LLIN campaigns, aiming to quickly increase coverage of specific target groups. National stakeholder meetings (including NMCP, donors and implementing partners) were held to design a generic national campaign strategy. Each campaign was then based on this national model, but detailed implementation plans were developed by the implementing partners, taking account of the distribution setting and lessons learnt from previous campaigns. The two campaigns examined in this study used a similar distribution model (Table 2). Implementation was led by Malaria Consortium and the NMCP, and was conducted over a period of one month in March 2007 (Adjumani district) and September 2007 (Jinja district); the target groups were pregnant women and children under five years of age.

### ANC delivery

In January 2007, free public provision of LLINs to pregnant women attending ANC clinics was launched in Adjumani district (Table 1). During a two-day training workshop, all ANC service providers (midwives and nursing assistants) were provided with refresher training on malaria and its prevention, and trained on the practical aspects of the LLIN delivery, data management and IEC. From then on, pregnant women attending their first ANC visit received an LLIN and were provided with expanded health education sessions including additional information on the risks of malaria in pregnancy, the benefits of LLINs and a practical demonstration on how to hang and use the net.

LLINs allocated to ANC distribution had been stored at Malaria Consortium premises in Kampala and been transported to the district by a sub-contractor. At district level the MoH store was used and the DHT was responsible for providing transport and maintaining a supply of LLINs to the health facilities. Malaria Consortium staff provided support to the DHT in managing the distribution system and conducted regular monitoring visits to facilitate the integration of ANC-based LLIN delivery into routine MoH operations.

### Collection of quantitative data

The household survey used a two-stage cluster sampling design. First, campaign distribution points were randomly selected from a list of all distribution points in Adjumani or Jinja. For study of the ANC distribution, two sub-counties in Adjumani (Cifro and Adrope) were pre-selected, as these had not benefitted from campaign distributions. Within these sub-counties, ANC outlets were randomly selected, excluding those that had distributed less than 50 LLINs in January and February 2007 to avoid including facilities that had experienced serious stock-outs during this period. At the distribution points, net recipients were selected at random from either the ANC registry book or, in the case of campaigns, from the distribution register. Households of net recipients where then visited by the survey team in their villages and invited for an interview. A piloted, pre-coded questionnaire was developed to collect household data on LLIN retention and use, and to collect data on the net recipients' knowledge of malaria and their socioeconomic characteristics.

### Analysis of quantitative data

The data were double entered using EpiData 3.1 software ("The EpiData Association" Odense, Denmark) and compared to check for completeness and conflicting entries. Final analysis was conducted in STATA 9.0 (Stata Corp., USA 2005). All analyses were carried out with adjustment for the cluster design using the "svy" command family in STATA. An LLIN was considered to have been retained if it was either verified by the interviewer as being of the distributed LLIN brand or if sufficient detail was provided by the household to make it plausible that the net existed (in those cases where households refused access to the home). An LLIN was considered to be in use if any person in the household had reportedly slept under it during the previous night. A wealth index was calculated based on household assets and house qualities using principle component analysis. Wealth quintiles were then constructed separately for each survey site. Standard errors and confidence intervals for the concentration indices were calculated using the spreadsheet developed by the World Bank based upon the formula by Kakwani *et al *for grouped data [20].

### Collection of cost data

The cost analysis adopted the provider perspective, which was defined as the costs incurred by Malaria Consortium and the MoH. This meant that costs to LLIN users, such those associated with travel to and waiting at distribution points, were not captured [21]. The cost components of each delivery strategy were identified using an ingredients approach, whereby all provider resources required to enable each of the LLIN distributions were valued [22] and organized into eight cost categories (Additional file 1). All costs associated with research activities were excluded.

Cost data were collected retrospectively, using financial expenditure records to capture financial costs, and financial as well as other sources of information to capture economic costs. Economic costs consisted of donated services and other items not covered by cash expenditure that were required to enable LLIN distribution. Noticeable differences between financial and economic costs were observed for the project management category, where ANC delivery was largely supported by drawing on spare capacity of Malaria Consortium staff, hence incurring limited financial cost, while both campaigns were supported by dedicated staff paid for by the projects.

Costs were measured in a combination of Ugandan Shillings (UGX) and United States Dollars (USD), depending on which currency was used to purchased or pay for the resources. Costs in UGX were measured at the time the expenditure was incurred and converted to USD 2007 prices, based on the average exchange rate for the year (1 USD = 1741 UGX;http://www.oanda.com). No adjustment for inflation was undertaken, as all costs were incurred during 2007. Items with a lifespan of more than one year were classified as capital costs and were annuitized using a discount rate of 3%, to be consistent with the recommendations of the World Bank [23]. Based on another costing study recently conducted in Uganda [24], the lifespan of cars was estimated at 7.5 years, that of motorcycles at four years and that of computers at three years. For both LLIN products, it was decided to use an average life expectancy of three years to calculate the number of TNYs generated by each intervention. Although some studies report or assume that Olyset^® ^nets are more durable [25] and longer life expectancies have been used in other costing studies [26], observations from an ongoing longitudinal study on LLIN durability in Uganda do not support such assumption or observation (Kilian *et al*, unpublished). The recent World Health Organization Pesticide Evaluation Scheme's 13^th ^Report also concludes that data are insufficient to assume that Olyset^® ^nets have an average life span of five years [27]. To be consistent with average Malaria Consortium overheads in Uganda, an 18% overhead was included for all ingredients other than capital items, while an 5% overhead was applied to the latter.

For each delivery system, the total project cost per LLIN (i.e. cost of all ingredients) and the delivery cost per LLIN (i.e. total cost per net excluding the purchase cost of the mosquito net) was calculated. Calculations of 'per LLIN' costs under each distribution was based on the total number of LLINs recorded as distributed for each system (for each campaign and during the January - December 2007 period for the ANC distribution). No adjustment was required to account for any loss of LLINs within the supply chain as all nets sent to the two districts were accounted for in the distribution records.

### Effects

Historically, mosquito net distribution programmes commonly reported the number of nets and insecticide treatments distributed [26]. Some studies aggregate these into a single unit of output, the treated net year (TNY), to facilitate comparison between settings. With the advent of LLINs, more recent work generally only provides the total number of nets delivered because, unlike traditional ITNs, the treatment is expected to last the lifetime of the net. To produce an output measure that can be compared across studies using different ITN products, the number of TNYs from LLINs distributed can be expressed as the total number of LLINs multiplied by the expected life of the net in years [9,28,29]. For the present study both the cost per LLIN delivered and the cost per TNY are reported. To account for the fact that not all LLINs distributed are retained or regularly used by target populations, the retention and use results generated by the present study were incorporated into the cost analysis, thus estimating the cost of delivering an LLIN to the end user.

### Sensitivity analysis

The cost calculations involved a number of assumptions. To explore how the results responded to changes in these assumptions, one-way sensitivity analyses (i.e. varying one parameter at a time) was used. As recommended elsewhere [26], the discount rate (0% - 10%), the life expectancy of LLINs (2 - 5 years) and the cost of a LLIN were varied. The latter was decreased for all distribution channels, because increasing competition in the LLIN market has led to a decrease in the cost of both the PermaNet^® ^and Olyset^® ^products distributed here. Cost estimates of USD 3.9, USD 4.5 and USD 5.5 used in the sensitivity analysis were based on recent quotes obtained by Malaria Consortium for a large volume of polyester nets, a large volume of polyethylene nets, or a small volume of polyethylene nets, respectively.

### Collection and analysis of qualitative data

To better understand the determinants of LLIN retention and use, a 'topic guide' was developed to facilitate semi-structured focus group discussions (FGDs) and interviews. The topic guide was piloted with health workers in Adjumani and subsequently refined to aid comprehension. Male and female participants were recruited and allocated to one of three groups: 1) neither received an LLIN nor information about its use, 2) received an LLIN and information, or 3) had received an LLIN, but either did not use it or had not retained it. Ten FGDs and twelve interviews were conducted in the local language through translators in Adjumani, while ten FGDs and five interviews were conducted in Jinja. Interviews, rather than group discussions, were conducted with individuals from group 3, because the information to be discussed was considered sensitive and a group setting thought to be unlikely to elicit the desired level of detail. FGDs and interviews continued until little new information emerged [30]. English language transcriptions of FGDs and interviews were read repeatedly and analysed thematically using the 'framework approach' [31]. The analysis was conducted by one researcher with no known conflict of interest.

### Ethical considerations

The study was conducted according to the principles of the declaration of Helsinki and the international guidelines of biomedical research involving human subjects [32]. Ethical approval was granted by the Uganda National Council for Science and Technology. The study was explained to the head of each of the selected households, who was then asked to provide written consent to participate in the study. Households that did not provide consent were excluded from the study.

## Results

### Quantitative data

#### The sample

A total of 1,614 net recipients were sampled from the distribution registers of which 1,445 were successfully followed up and completed interviews. The major difference between the two distribution channels was that 9.8% of sampled recipients from the ANC distribution were unknown in the community while all LLIN recipients from campaigns were verified as residents. It was assumed that these 'unknown' recipients entries were generated either by health facility staff who used the nets otherwise or by women who used a false name to obtain additional nets. Another difference was the level of mobility in Adjumani, with 8% (ANC) to 10% (campaign) of recipients having moved away since the distribution, while no such case was reported from Jinja.

There were considerable socio-economic and demographic differences between the two districts, but also between the ANC and campaign area within Adjumani district (Table 3). The level of awareness about malaria transmission and means of protection was generally high, but significantly higher in Adjumani compared to Jinja.

**Table 3:** Demographics and net distribution and ownership (95% confidence intervals in parenthesis)

Variable	ANC Adjumani	Campaign Adjumani	Campaign Jinja	p value	Comparison
**Households interviewed**	**N = 378**	**N = 520**	**N = 547**		

Household members *Mean (95% CI)*	5.7 (5.3-6.1)	5.8 (5.6-6.0)	6.5 (6.3-6.8)	<0.0001	Adjumani vs. Jinja

Children under five years per household *Mean (95% CI)*	1.8 (1.7-1.9)	1.8 (1.6-2.0)	2.0 (1.9-2.2)	0.005	Adjumani vs. Jinja

Sleeping places *Mean (95% CI)*	2.3 (2.1-2.5)	2.1 (1.9-2.3)	3.0 (2.7-3.3)	<0.0001	Adjumani vs. Jinja
Persons per sleeping place *Mean (95% CI)*	2.7 (2.5-2.8)	2.9 (2.7-3.3)	2.5 (2.3-2.7)	0.02	Adjumani vs. Jinja
**Before distribution**					
Households with any net previous to distribution *Percent (95% CI)*	42.6% (32.7-52.5)	27.3% (16.1-38.6)	21.9% (16.6-27.3)	0.04	ANC vs campaign Adjumani
Households with net to sleeping place ratio ≥ 1 before distribution *Percent (95% CI)*	7.4% (3.8-11.0)	4.4% (1.8-7.0)	4.4% (1.5-7.2)	0.15	ANC vs campaign Adjumani
Households with persons to net ratio of ≤ 2 before distribution *Percent (95% CI)*	4.7% (2.6-6.9)	2.5% (0.7-4.3)	2.9% (1.0-4.9)	0.11	ANC vs campaign Adjumani
**Immediately following distribution**					
LLIN distributed per household* Mean (95% CI)	1.0 (n.a.)	1.48 (1.36-1.60)	1.83 (1.69-1.98)	<0.0001	Campaign Adjumani vs. Jinja
Households with net to sleeping place ratio ≥ 1 better after distribution *Percent (95% CI)*	37.0% (26.8-47.2)	60.4% (48.7-72.1)	47.2% (36.6-57.8)	0.08	Campaign Adjumani vs. Jinja
Households with persons to net ratio of ≤ 2 after distribution *Percent (95% CI)*	26.7% (20.2-33.2)	28.7% (20.7-36.6)	34.0% (27.9-40.1)	0.25	Campaign Adjumani vs. Jinja
**At time of survey**					
Households that obtained additional nets after distribution *Percent (95% CI)*	15.6% (9.0-22.2)	9.2% (5.4-13.1)	2.7% (0.5-4.2)	<0.0001	Campaign Adjumani vs. Jinja
Nets owned per household* Mean (95% CI)	1.68 (1.51-1.84)	1.94 (1.70-2.17)	2.21 (2.03-2.40)	0.04	Campaign Adjumani vs. Jinja
Households with net to sleeping place ratio ≥ 1 *Percent (95% CI)*	44.7% (34.4-55.0)	65.8% (55.2-76.8)	49.0% (38.2-59.8)	0.025	Campaign Adjumani vs. Jinja
Households with persons to net ratio of ≤ 2 *Percent (95% CI)*	35.5% (24.3-38.7)	32.1% (23.7-40.5)	37.5% (32.1-42.8)	0.26	Campaign Adjumani vs. Jinja

* proportion of households with at least one net/ITN was 100% in all groups

#### Net ownership

Pre-distribution household coverage with any net was slightly higher in Adjumani than Jinja (33.7% vs. 21.9%), but the proportion of households that had all sleeping places covered or a ratio of one net for every two people (universal access) was equally low in all three sites (Table 3). The average number of LLIN received during the campaigns was higher in Jinja than in Adjumani, reflecting the larger family size, but when the persons-to-net ratio was considered, no significant difference in outcome between the campaigns could be detected.

#### Equity of distribution

**Figure 1:**
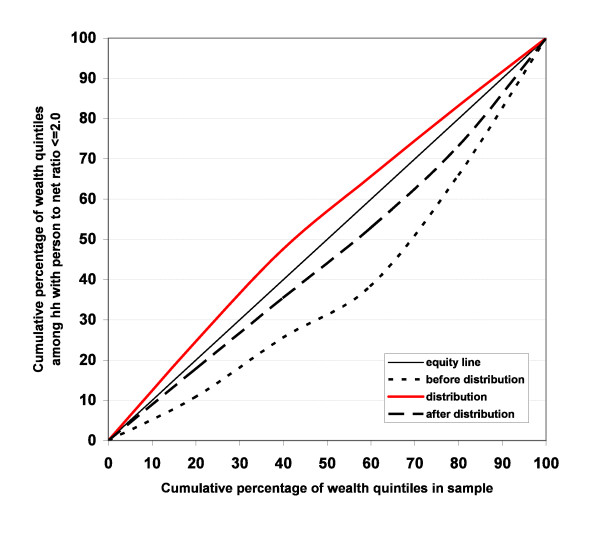
**Equity of household net ownership**. Concentration curves for proportion of households with a person to net ratio of 2.0 or better based on nets received from free public distribution (red line) and before (dotted) and after (dashed) distribution considering all nets in household.

Household ownership of any nets before the public free distributions showed a similar poor-to-wealthy gradient in all three study areas, with a concentration index of 0.19 (95% CI 0.11-0.26). Since all sampled households had at least one LLIN after the distribution, the proportion of households with a person to net ratio of 2.0 or lower was chosen as the criteria for equity evaluation. The before-distribution concentration index was very similar to that for "any net" with 0.24 (0.18-0.29). For the LLIN distributed through ANC or campaign the index was -0.09 (-0.11- -0.06) indicating a significant pro-poor distribution. This led to a much improved index of 0.08 (0.02-0.15) after the distribution when considering all nets owned by the households (Figure 1).

#### Net retention

Retention rates of LLINs received through the distributions were generally very high (Table 4). Surprisingly, retention in Jinja was slightly lower than in Adjumani, even though the survey took place two months earlier after distribution. In the case of the ANC distribution, retention rate reduced to 88.0% (95% CI 79.2-96.9) if the 48 suspected fraudulent register entries were included as non-retained nets. This would reflect a more programmatic analysis representing the proportion of nets delivered to the health facility that were in possession of the target group at the time of the survey.

**Table 4:** Retention and use of nets (95% confidence intervals in parenthesis)

Variable	ANC Adjumani	Campaign Adjumani	Campaign Jinja	p value Campaign Adjumani vs. Jinja
**Retention (nets)**	**N = 378**	**N = 772**	**N = 1003**	

Mean time since distribution in months	7.3 (7.1-7.4)	7.5 (7.4-7.5)	5.1 (5.0-5.1)	<0.0001

LLINs retained by recipient household	99.2% (98.5 - 99.9)	98.3% (96.4-100)	93.6% (91.6-95.7)	0.019

**Retention (households)**	N = 378	N = 520	N = 547	

Households with all nets retained	- -	97.5% (92.9-99.2)	90.5% (87.7-92.7)	0.017
**Use (nets)**	N = 375	N = 759	N = 939	
LLINs retained that were slept under the previous night	98.9% (97.2 - 100)	97.0% (94.3-99.6)	74.2% (74.2-81.5)	<0.0001
**Use (households)**	N = 378	N = 520	N = 547	
Households where all U5 slept under a net the previous night	93.9% (89.5-96.5)	92.8% (85.8-96.5)	56.4% (52.1-60.5)	<0.0001
Households where all members slept under a net the previous night	29.6% (22.9-37.4)	42.7% (35.3-50.5)	18.5% (13.7-24.5)	<0.0001
Households with net to sleeping place ratio of ≥ 1 where all members slept under a net the previous night	55.6% (45.7-65.1)	57.9% (51.1-64.4)	32.5% (22.9-43.8)	0.0009
Households with person to net ratio of ≤ 2 where all members slept under a net the previous night	62.2% (54.7-69.2)	73.7% (64.6-81.1)	39.0% (29.0-50.1)	0.0001

Of the 80 non-retained nets, the majority (50) were reported to have been given away; 38% in Adjumani and 68% in Jinja (p = 0.05); other nets were reported as destroyed, stolen or thrown away because of holes; in 17 cases (21%) no reason was given. In order to evaluate the determinants of non-retention, "net given away" was focused on, as this was clearly a behaviour driven outcome. Including all nets from the distributions (n = 2153) a multivariate logistic regression analysis showed that significant factors preventing a net from being given away were: i) belief of the head of households that nets prevent malaria (Odds-Ratio 0.34, 95% CI 0.24-0.48, p =< 0.001), ii) the number of children under five in the household (Odds Ratio 0.61, 95% CI 0.43-0.86, p = 0.006), and iii) ownership of nets before the distribution (OR 0.42, 0.18-0.99, p = 0.05). Factors that lowered the likelihood of retention were: i) having more than one net per sleeping place (OR 3.11, 1.44-6.73, p = 0.005), ii) secondary or higher education of head of household (OR 2.25, 1.13-4.47, p = 0.22), and iii) coming from Jinja district (7.14, 2.31-22.06, p = 0.001). Socio-economic status (wealth quintiles), having received information about net use or a demonstration on the use of the net had no significant impact on retention.

#### Net use

The difference between Adjumani and Jinja districts regarding LLIN use was greater than that for retention (Table 4). This variable did not differ between delivery channels within Adjumani. Looking at all family nets, the most influential factor associated with net use was if the net was from one of the public distributions studied. Other associations with use of nets were large family size (more than seven) and having received information and a demonstration on net use (Table 5). A strong inhibitive factor for net use was oversupply, defined as net to sleeping place ration of more than 1.0; the second most inhibitive factor was the respondent reporting difficulties in using the net.

**Table 5:** Determinants of net use. Results of multivariate analysis (N = 2745).

Variable	Odds-ratio	95% Confidence interval of OR	p-value
Order of nets in household			
first	1.00	- -	
second	0.38	0.27 - 0.52	<0.0001
third	0.16	0.10 - 0.23	<0.0001
fourth	0.34	0.13 - 0.87	0.025
fifth	0.04	0.00 - 0.85	0.039
Source of net			
free or subsidized outside distribution	1.00	- -	
distribution (ANC or campaign)	8.39	3.98 - 17.74	<0.0001
commercial sector	1.69	0.78 - 3.64	0.18
Household is oversupplied			
net to sleeping place ratio >1.0	0.44	0.33 - 0.59	<0.0001
Distribution			
ANC Adjumani	1.00	- -	
campaign Adjumani	0.35	0.21 - 0.57	<0.0001
campaign Jinja	0.09	0.05 - 0.15	<0.0001
Net was obtained before ANC/campaign	1.78	0.94 - 3.38	0.075
Household members 7 or more	1.43	1.06 - 1.92	0.020
Children under five years in household are 5 or more	1.93	0.91 - 4.11	0.085
Household received information on hanging plus demonstration	1.44	0.98 - 2.11	0.062
Household reported to have had difficulties in hanging or using nets	0.63	0.40 - 0.99	0.048
Household is from 2 lowest wealth quintiles	0.75	0.51 - 1.11	0.15

Within the same family not all nets are used in the same way and an interesting finding of this study is that that there was a tendency for respondents to present the most frequently used nets first when asked about other household nets. A factor affecting use or non-use was shown to be what "number" the net had been recorded as on the household questionnaire, with net 1 more likely to be in use than net 2 etc (Table 5). This finding was independent from the number of nets the family owned and observed in all three surveys.

The variables defining the distribution channel and distribution site remained significant after controlling for the other explanatory variables. This indicates that factors other than those captured in the survey significantly determined use of nets. Other variables were tested but dropped from the model due to lack of association.

The mean number of people sharing a net was 2.5 for the ANC survey population, 2.6 for the Adjumani campaign survey and 2.1 for Jinja. The difference between Jinja and Adjumani was statistically significant (p < 0.0001).

There were significant differences between the channels studied in terms of who used the nets. In the case of the Adjumani ANC delivery channel, 76.3% of the 595 used nets were shared between mother and young children and 10.6% were used by children only. In contrast, for the Adjumani campaign distribution these proportions were 71.2% and 21.6% (n = 861), while they were 42.2% and 44.8% (n = 784) for the Jinja campaign. The difference within Adjumani (p < 0.0001) could be due to the fact that beneficiaries of the ANC delivery channel had fewer nets per household (Table 3), while the difference between Adjumani and Jinja (p < 0.0001) seemed to be a different behavioural pattern in the users.

The way in which the nets were used by families varied between study sites and, as with the likelihood of use, with the order in which the nets were presented to the interviewer. This was the case both for the number and type of people sleeping under each net. For example, the nets first presented were more likely to be used by more people with progressively fewer users for subsequently presented nets (P < 0.0001); and the nets first presented were predominantly used by a mother and child (78.4%); with subsequently presented nets more likely to be used by other adults. These findings were independent of the number of nets owned and was seen in all three surveys.

### Total financial and economic costs

The total financial cost of delivering LLINs through ANC services in Adjumani was USD 100,122, compared to USD 115,956 and USD 80,452 for campaigns in Adjumani and Jinja, respectively. Total economic costs were considerably lower, at USD 66,618, USD 58,092 or USD 37,467 for the same three distributions. The substantially lower economic costs can largely be explained by the fact that the cost of purchasing LLINs, which made up 81 - 85% of the total financial cost, was annuitized over an assumed useful life of three years. For PermaNet^®^, which had been purchased at a cost of USD 5.26 each, annuitization resulted in an economic cost of USD 1.86 per year, while the equivalent for Olyset^® ^was USD 5.75 annuitized to USD 2.03 (Additional file).

### Outputs and cost-effectiveness analysis

During 2007, a total of 15,188 PermaNet^® ^were delivered through ANC services in Adjumani, while 16,378 Olyset^® ^and 12,994 PermaNet^® ^were distributed through campaigns in Adjumani and Jinja, respectively. Financial and economic costs per LLIN delivered (excluding the cost of the net) were highest for the ANC channel and lowest for the Jinja campaign (Additional file 2).

Differences in cost per LLIN delivered through the three channels were predominantly a result of the varying financial/economic contributions to project management. The economic cost (excl. cost of the net) per LLIN delivered through ANC was more than twice the financial cost, as it accounted for the contributions of Malaria Consortium staff whose time was paid for by other projects.

The proportion of costs accrued by each cost category varied considerably between two distribution channels (ANC or campaign), but also between the two campaigns. The most costly component of ANC delivery was transportation of LLIN some 500 km from Kampala to Adjumani. For campaign distribution in the same district, transport still constituted a considerable part of the total economic cost (14.7%), but by far the most costly activity was LLIN distribution within the district. Economic distribution costs also constituted a large proportion (20.4%) of the overall costs of the campaign in Jinja, but were superseded by the cost of sensitization and IEC (21.6%).

In general, the cost per LLIN delivered or per TNY only increased moderately when these effects were adjusted using actual retention and/or use of LLINs by the target groups (Additional file 2). However, a 45% increase in the cost per LLIN delivered (i.e. USD 1.10 *vs *USD 0.76) was observed after adjusting results for the Jinja campaign for the relatively low retention and use.

### Sensitivity to underlying assumptions

For both the cost per LLIN delivered and the cost per TNY, the implications of varying the underlying assumptions were explored, showing that the cost estimates were most sensitive to variations in the assumed life expectancy of the LLIN (Additional file 3). The cost per TNY almost doubled when life expectancy was decreased from three to two years, with a similar but less pronounced effect observed for the economic cost per LLIN delivered.

### Results of qualitative investigation

Focus groups and discussions with individuals showed that people generally understood that LLINs are effective in preventing malaria infection. While different LLIN products and locally made nets were mentioned in discussion, LLINs were widely considered as more desirable.

*I prefer the treated nets because if one sleeps in the untreated nets a mosquito can come near the net and it keeps shouting and making noise and sometimes you may even think the mosquito has found its way in. For the treated nets the mosquitoes die immediately*.

(Woman interviewed in Adjumani)

Appropriate techniques of use were largely understood, although exposure to relevant information and its comprehension varied considerably between respondents. Prioritization of who was to sleep under a mosquito net in households with insufficient numbers to cover all inhabitants also varied. Identity and role within the family and broader community, and the status afforded as a result, were clearly some of the determinants of who was sleeping under a net.

*When he asked me for it I asked him what I would be using and he told me that for me I will get another one and then I am obliged to give to him because he is my brother. Brothers in our culture are respected*.

(Woman interviewed in Jinja)

Discussions around reasons for non-use of LLINs and other types of mosquito nets owned by households centred on concerns (often formed from hearsay) about discomfort and 'illness' from using certain types of nets, as well as challenges in keeping them clean and free of holes. Commonly these challenges related to living conditions.

*These nets are too white and that may prevent people from using them. They easily show the dirt and yet we stay in a dusty environment*.

(Woman in FGD in Jinja)

Participants of focus groups and interviews were often reluctant to openly discuss reasons why LLINs were not retained. Respondents generally felt that the donated LLIN should have been retained by recipients and appeared to assume that the interviewer or leadership figures would disapprove of people having sold or given away their LLIN.

There was considerable variety and depth in the explanations for not retaining the LLIN. Theft and sale were reported occasionally, while physical damage to the net was the most commonly stated reason why people were no longer in its possession.

*... they sleep on a papyrus mat and the edges are constantly destroyed by the papyrus mat*.

(Woman in FGD in Adjumani)

The apparent reluctance of respondents to discuss reasons for non-retention made it unlikely, however, that the reported reasons provide a full explanation for non-retention observed in the present study.

## Discussion

The present study measured retention, use and delivery costs for LLINs distributed through ANC services and campaigns in parts of Uganda, and investigated why some beneficiaries did not retain or use their net. The work was conducted to contribute to the evidence-base on using these two delivery channels in Uganda. Reassuringly, the data show that LLIN delivery through either channel can achieve high levels of retention and use around six months after LLIN distribution, with similar financial investment. However, taking in-kind contributions by the provider into account showed that ANC delivery was nearly twice as expensive as campaigns.

### Retention and use

The high retention values recorded six months after distribution were at least as good as findings from other parts of Uganda and elsewhere. Follow up of LLINs distributed in 2004 through ANC facilities in the conflict affected district of Kitgum, Uganda, found that 86% of recipients were still in possession of the net after an average period of two and a half months [33]. Similarly, one year after distribution of ITNs to the internally displaced persons (IDPs) in Western Uganda, 80% of nets had been retained [14]. A recent study in Ethiopia reported 72% retention after two to three years [34]. That retained nets are used, however, cannot be assumed.

Encouragingly, the results showed that almost all of the LLINs that were retained were also reportedly in regular use. This finding is in stark contrast with that of the above mentioned work in Kitgum, Uganda, which initially found that only 39% of retained nets had been used by pregnant women during the previous night [33]. Through concerted IEC activities it was possible to increase use to 89% [35], which is comparable to the present findings. It is not uncommon to see lower levels of use compared to ownership or retention. Results from follow-up surveys six-months after ITNs were distributed alongside vaccination campaigns also reported rather low use by children under five; 60% from Ghana [18] and 56% from Zambia [17]. In the Western Uganda IDP study 57% of household members said that they slept under their net the previous night [14], whilst in the Ethiopian study 62% of respondents claimed that their children slept under an LLIN [34]. In Eritrea 83% of households with ≥ 1 ITN reported that all children under five had slept under a net the previous night [36].

In closely controlled trial settings both retention and use rates are often higher than in programmatic environments [25,37-39], possibly due to more intense initial IEC and better follow-up. For example: 92% of Afghan refuges in a trial in Pakistan still had their ITNs two years after distribution [13]. Use rates after large scale ITN trials were 73.5% in the first and 67.5% in the second year in Kenya [38], 70% after three years in Tanzania [39], 87% after one year in Cambodia [40], and as high as 94% seven years after distribution in a small scale project in Tanzania [25]. The data from the present study is consistent with the above findings from Eritrea [36], demonstrating that even under programme conditions it is possible to achieve high use rates six months after distribution.

To improve on LLIN use, "hang up" activities are increasing proposed as a standard and crucial part of campaign distributions [41]. Such activities require volunteers to go from house to house after the campaign, promoting LLIN use and helping families with hanging of the net. Positive outcomes in terms of use have been reported following "hang up" activities in Togo. Nine months after distribution 80% of people were using ITNs in households having had "hang up" visits compared to 72% in households that had not received this support [42]. In Niger, however, no such effect was observed for use of nets by children under five when households that had/had not received a post-campaign hang up visit were compared [16].

Given the multitude of sources of influence on net use it is not possible to accurately determine the specific effect of the IEC and BCC interventions used here. The two campaigns evaluated in the current study differed in their post distribution BCC activities. While Adjumani did not benefit from any activities, community volunteers in Jinja were asked to continue awareness raising on the importance of regular LLIN use after distribution and to conduct follow-up visits to recipient households to discuss use and assist with hanging of nets. It is not known to what extent these suggested "hang-up" activities took place, as no supervision or monitoring visits were conducted after the campaign. Additional exposure to post-distribution messages on LLIN use is likely to have take place through other communication channels. In Adjumani, BCC messages delivered as part of on-going LLIN delivery through ANC facilities may have acted as a route of on-going messaging, ensuring that the importance of net use was repeatedly reinforced. In Jinja, the active commercial sector may have provided information on the value and benefits of LLINs to the community at large. Whilst the relative importance of the different communication channels and BCC messages is unclear, it is evident that an elaborate house-to-house "hang up" activity was not necessary to achieve high use.

### Measuring retention and use

The proportion of nets retained and used in any given project will likely fluctuate and gradually decline over time. The present study examined LLINs distributed around six months previously; other studies often select other time points after distribution. An alternative approach to collection data, with a focus on data needed to inform programming, may be to avoid relatively labour intensive retention and use surveys that generate specific proportions and rather generate more frequent snapshot estimates of use, for example through use of the lot quality assurance sampling technique [43]. This method has been successfully used for rapid schistosomiasis assessments in Ugandan schools [44], which may also provide a suitable surveillance platform for malaria, including assessments of LLIN coverage [45]. Data collection would have to be conducted at different points in time after distribution (and at different times of the year), to determine whether follow up BCC and/or practical hang up support activities are required to encourage people to sleep under nets.

While the indicator for the assessment of minimal net coverage is well established as "proportion of households with at least one net or ITN" [46], there is as yet no agreement on how best to measure universal coverage. The two possible indicators used in this study to assess the intra-household coverage with nets are "proportion of households with at least one net per sleeping space" and "proportion of households with at least one net for every two people". There was, however, a significant difference between the two (Table 3), due to the fact that the mean number of persons per sleeping place in this study population deviated from the value 2. This indicates that persons to net ratio may be the better variable to use to measure universal coverage. Eisele and co-workers have used a similar indicator, namely the mean ratio of people to ITN in ITN owning households as a measurement of "universal access" [47]. This indicator is very similar to the proportion of households with a ratio of people to ITN ≥ 2.0, with the difference that the proportion is independent of the distribution of values and therefore more robust, making it the preferable indicator.

### Costs and implications for scale-up

Comparing LLIN delivery costs between studies is complicated by lack of consistency between the methods used [26]. Taking this caveat into account, the costing results were only compared to studies that used the same perspective (provider) and included the same or similar cost categories.

For campaign distribution, the financial costs of USD 6.19-7.08 (including the LLIN) estimate here were similar to the cost of USD 5.95 estimated for LLIN delivery alongside an Integrated Child Health Campaign in Togo [48], but nearly twice as high as that of USD 3.74 for delivery conducted alongside a vaccination campaign in Ghana [18]. The estimated marginal delivery cost alongside the vaccination campaign was USD 0.32, compared to the present estimated delivery costs (excluding the LLIN) of USD 0.67 - 1.04. These higher cost for stand alone campaigns in Uganda highlights the potential to reduce costs through integration of LLIN distribution with other public health campaigns, including those for control of neglected tropical diseases [49].

The delivery cost (excluding the LLIN) estimated here were similar for the two distribution channels studied in Adjumani (just over USD 1.0 per net delivered), and consistent with the USD 1.0 per person estimated in 2003 to cover the cost of scaling up ITN coverage in sub-Saharan Africa [50]. Importantly, the cost of campaign delivery was considerably higher in Adjumani when compared to Jinja (USD 1.04 *vs *USD 0.67), due to factors such as distance from Kampala and the number of sub-counties targeted. This highlights that both these factors need to be taken into account when budgeting LLIN distributions elsewhere in Uganda.

Average economic costs of USD 2.63 per ITN distributed or USD 4.41 per TNY have been reported for a social marketing program in Malawi [29]. Somewhat lower values have been calculated for the cost per TNY for LLINs delivered through public health services in Eritrea (USD 1.4) or through campaigns in Togo (USD 1.9) [9]. The latter study assumed that mosquito nets and their insecticide treatment lasted three years and is hence directly comparable with the range of estimates reported here. To compare the present results to those from Malawi, it would be necessary to adjust the expected life of a net to five years, leading to considerably lower costs per TNY, ranging from USD 0.43 for the Jinja campaign to USD 0.74 for distribution through ANC in Adjumani.

Once all of the provider inputs were take into account, the resulting economic costs showed that ANC delivery was almost twice as expensive as campaign delivery in Adjumani. The most important economic cost was staff time provided by Malaria Consortium and the Ugandan MoH. For the distributions studied, these additional costs were not a matter of concern, as both organizations had spare capacity to support ANC delivery. Nevertheless, information about these different components is important to plan and budget scaling up of ANC-based LLIN delivery. However, as routine LLIN distribution systems are scaled up, the amount of supervision and input required from implementing partners is likely to reduce over time. In this example of ANC distribution the economic costs, here inflated by considerable personnel time required during start-up activities, will likely move closer to the financial costs as the distribution system becomes institutionalized.

As discussed above, recent costing studies for LLIN delivery channels have reported the cost per net delivered and/or the cost per TNY. The latter are calculated on the assumption that the total number of nets delivered will be used over an average "useful life of a net", i.e. the number of years that a mosquito net physical lasts. In reality, however, the average "useful life of a net" is determined not only by the condition of the net, but also by the availability of replacement nets, among other factors. Mosquito nets with a few holes that would be considered to be in good condition in settings where new nets are scarce may be readily replaced by households regularly receiving free campaign nets or wealthy enough to buy new ones in the market. In addition, LLINs that are not used regularly will not provide the full amount of TNYs to the user. To reduce the assumptions underlying these cost-effectiveness estimates, this paper describes a modified approach. As well as conventional TNYs, TNYs linked to proportions of nets retained and in use approximately six months after distribution were calculated, and these were then used to adjust the cost estimates. These modified estimates are more accurate and it is hence proposed that such calculation should complement conventional cost estimates in situations where net tracking surveys or other means of estimating retention and use can be implemented. Similarly, the cost per LLIN delivered should also be adjusted using retention and use data, as applied here.

### Limitations of the study

The present study had two key limitations. First, direct comparison between our results and those of other studies was complicated by the fact that retention and use estimates are only point estimates and are likely to be affected by when they are being generated. Retention will decline gradually over the estimated three-year life of the LLIN while use will likely fluctuate, due to factors such as initial interest in a new product, IEC/BCC activities and seasonality [10,16,51-53]. Similar campaign approaches in the same country may thus seem to vary in their effectiveness of getting recipients to sleep under nets, depending on when this indicator is being measured. For the surveys conducted here this may be a reason for the differences in use between Jinja and Adjumani. Seasonality cannot be clearly measured as an influencing factor on use of nets with this study design. However, it was noted that the Jinja follow-up survey, which saw significantly lower use of nets than reported from Adjumani, was conducted in the dry season (end January - early February), whereas the Adjumani surveys were conducted at the end of the rainy season (October). Secondly, retention and use of LLINs and the cost of delivery are affected by a wide variety of context-dependent factors, such as specific population characteristics or access to health services. This means that the present results cannot be readily extrapolated to other locations in Uganda.

## Conclusions

Achieving impact on malaria morbidity and mortality in sub-Saharan Africa will require considerable improvements in the availability and use of effective malaria prevention tools - most importantly LLINs. The Roll Back Malaria (RBM) Partnership aims at 80% use of ITNs and other appropriate vector control methods by 2010 [3]. In Uganda, a mixed-model of campaign and ANC delivery channels is used to deliver LLINs free-of-charge in an attempt to reach this target. Encouragingly, it was found that both approaches resulted in high LLIN retention and usage, exceeding the RBM Partnership's targets for the study population. Campaigns are logistically demanding and thus unlikely to be a practical solution to ensuring that LLIN coverage remains high over time. Uganda's ANC services seem to provide a suitable complement to keep up LLIN coverage of pregnant women, although at slightly higher economic cost. Additional delivery channels will need to be explored so that all populations groups have access to and use LLINs regularly. Costing of these channels, as well as ANC and campaign distribution at larger scale than in the present study, will be needed both to inform budgeting of the national strategy and to ultimately determine its cost-effectiveness. To do so, the cost per net delivered and TNYs provide useful indicators for comparison of delivery channels, but their calculation should focus on outcome (use of nets by target groups), not just output (number of nets delivered) as practiced to date.

## Competing interests

The authors declare that they have no competing interests.

## Authors' contributions

JK, KK and AK conceived the study. DK, DS, TT, KK and JK designed the study components, with input from AK, and coordinated its implementation. AK, KK and NW analysed the quantitative data on net usage and retention, DS analysed the qualitative data, and JK analysed the cost data. JK, KK and AK led the preparation of the manuscript. All authors read and approved the final manuscript.

## Supplementary Material

Additional file 1 **Table of detailed cost data**. Details on inputs, quantities, and associated costs required to implement the LLIN delivery strategies (base-case scenario) described in the manuscript. Proportional allocation was used to calculate the quantities of each line item; this resulted in relatively small figures for some lines, as many activities targeted more districts than the ones studied. Sub-totals shown are based on actual figures, not the rounded ones shown for each line item. Click here for file

Additional file 2 **Table of costing results**. Financial and economic cost per LLIN delivered and cost per TNY. Results are reported both for an assumed 100% retention and use, and adjusted for actual retention and use as observed in the study (see Table 4). Adjusted costs were calculated by reducing the total number of nets delivered by the relevant proportions shown in Table 4 in the rows 'LLINs retained by recipient household' and 'LLINs retained that were slept under the previous night'. Click here for file

Additional file 3 **Analysis of sensitivity of cost estimates to assumptions**. Table showing results of one-way sensitivity analysis Click here for file
