# Supplementary material for: Costs and effects of two public sector delivery channels for long-lasting insecticidal nets in Uganda
Source: Malar J. 2010 Apr 20;9:102. doi: 10.1186/1475-2875-9-102 (PMC2868859; doi:10.1186/1475-2875-9-102)
Supplement: Additional file 1 — Table of detailed cost data. Details on inputs, quantities, and associated costs required to implement the LLIN delivery strategies (base-case scenario) described in the manuscript. Proportional allocation was used to calculate the quantities of each line item; this resulted in relatively small figures for some lines, as many activities targeted more districts than the ones studied. Sub-totals shown are based on actual figures, not the rounded ones shown for each line item. [file 1475-2875-9-102-S1.DOC]

Additional file 1: Details on inputs, quantities, and associated costs required to implement the LLIN delivery strategies (base-case scenario) described in the manuscript. Proportional allocation was used to calculate the quantities of each line item; this resulted in relatively small figures for some lines, as many activities targeted more than one district. Sub-totals shown are based on actual figures, not the rounded ones shown for each line item.

|  |  | ANC delivery – Adjumani district | | | Campaign delivery – Adjumani district | | | Campaign delivery – Jinja district | | |
| --- | --- | --- | --- | --- | --- | --- | --- | --- | --- | --- |
| Input | Unit | Quantity | Unit Cost (US$)^1^ | Cost Type^2^ | Quantity | Unit Cost (US$)^1^ | Cost Type^2^ | Quantity | Unit Cost (US$)^1^ | Cost Type^2^ |
| TRAINING: | | | | | | | | | | |
| *Central training*^3^ | | | | | | | | | | |
| Venue | Per day |  |  |  | 0.03 | 115 | F | 0.04 | 115 | F |
| Sound System | Per day |  |  |  | 0.03 | 43 | F | 0.04 | 43 | F |
| Water/Tea/Coffee | Per person |  |  |  | 2.43 | 3 | F | 3.33 | 4 | F |
| Buffet | Per person |  |  |  | 1.22 | 8 | F | 1.67 | 8 | F |
| Transport refund/allowance | Per person |  |  |  | 1.08 | 29 | F | 1.48 | 29 | F |
| Fees for Facilitator | Per day |  |  |  | 0.08 | 57 | F | 0.11 | 57 | F |
| Notebooks | Per person |  |  |  | 1.22 | 0.6 | F | 1.67 | 1 | F |
| Flip charts | Chart |  |  |  | 0.08 | 6 | F | 0.11 | 6 | F |
| Pens | Packet |  |  |  | 0.03 | 5 | F | 0.04 | 5 | F |
| Markers | Packet |  |  |  | 0.08 | 6 | F | 0.11 | 6 | F |
| Facilitators salary | Per day |  |  |  | 0.08 | 27 | E | 0.11 | 44 | F |
| Sub-total |  |  |  |  |  | 62 (60) | E (F) |  | 87 | F |
| *Refresher training* | | | | | | | | | | |
| Per diem participants | Per day | 62 | 20 | F |  |  |  |  |  |  |
| Transport refund participants | Per day | 31 | 5 | F |  |  |  |  |  |  |
| District trainers allowance | Per day | 2 | 29 | F |  |  |  |  |  |  |
| District trainers transport refund | Per day | 2 | 3 | F |  |  |  |  |  |  |
| Per diem for CDPO | Per day | 4 | 37 | F |  |  |  |  |  |  |
| Per diem, Malaria Consortium driver | Per day | 4 | 37 | F |  |  |  |  |  |  |
| Tea and lunch trainers | Per day | 2 | 4 | F |  |  |  |  |  |  |
| District trainers salary | Per day | 2 | 15 | E |  |  |  |  |  |  |
| Participants salary | Per day | 62 | 7 | E |  |  |  |  |  |  |
| CDPO salary | Per day | 4 | 90 | E |  |  |  |  |  |  |
| Malaria Consortium driver salary | Per day | 4 | 38 | F |  |  |  |  |  |  |
| Diesel, Gulu-Adjumani (return) | Per trip | 1 | 18 | F |  |  |  |  |  |  |
| Car purchase | Per day | 4 | 17 (15) | E (F) |  |  |  |  |  |  |
| Car insurance, tax & maintenance | Per day | 4 | 25 | E |  |  |  |  |  |  |
| Flip chart | Chart | 2 | 6 | F |  |  |  |  |  |  |
| Marker pen and masking tape | Packet | 1 | 5 | F |  |  |  |  |  |  |
| Note book, pens, bags | Book | 31 | 1 | F |  |  |  |  |  |  |
| Hall hire | Day | 1 | 57 | F |  |  |  |  |  |  |
| Sub-total |  |  | 3,056 (2,120) | E (F) |  |  |  |  |  |  |

^1^ Rounded to nearest dollar, if above USD 1

^2^ F = Financial, E = Economic

^3^ Proportional costs. Adjumani was part of one national workshop conducted for participants from 37 districts, while Jinja was part of one national workshop conducted for 27 districts.

|  |  | ANC delivery – Adjumani district | | | Campaign delivery – Adjumani district | | | Campaign delivery – Jinja district | | |
| --- | --- | --- | --- | --- | --- | --- | --- | --- | --- | --- |
| Input | Unit | Quantity | Unit Cost (US$)^1^ | Cost Type^2^ | Quantity | Unit Cost (US$)^1^ | Cost Type^2^ | Quantity | Unit Cost (US$)^1^ | Cost Type^2^ |
| TRAINING (continued): | | | | | | | | | | |
| *District training*^4^ | | | | | | | | | | |
| Transport refund, sub-county supervisors | Per person |  |  |  | 12 | 5 | F | 3 | 5 | F |
| Refreshments, sub-county supervisors | Per person |  |  |  | 12 | 3 | F | 3 | 3 | F |
| Central supervisors per diems | Per day |  |  |  | 3 | 37 | F | 1.5 | 37 | F |
| Central supervisor’s driver per diem | Per day |  |  |  | 1.5 | 37 | F | 0.5 | 37 | F |
| Central supervisors facilitation allowance | Per day |  |  |  | 2 | 17 | F | 1.5 | 17 | F |
| Venue | Per day |  |  |  | 1 | 14 | F | 0.5 | 14 | F |
| Salary, sub-county supervisor | Per day |  |  |  | 12 | 14 | E | 3 | 14 | E |
| Central supervisor’s driver salary | Per day |  |  |  | 1.5 | 18 | E | 0.5 | 18 | E |
| Car purchase cost | Per day |  |  |  | 1.5 | 17 (15) | E (F) | 0.5 | 17 (15) | E (F) |
| Car insurance, tax & maintenance | Per day |  |  |  | 1.5 | 25 | E | 0.5 | 25 | E |
| Fuel to district (return) | Per trip |  |  |  | 0.5 | 158 | F | 0.5 | 24 | F |
| Fuel within district | Per day |  |  |  | 1 | 11 | F | 0.5 | 11 | F |
| Sub-total |  |  |  |  |  | 656 (416) | E (F) |  | 221 (155) | E (F) |
| *District training of trainers* | | | | | | | | | | |
| District Health Education Officer | Per day | 1 | 18 | E |  |  |  |  |  |  |
| Malaria Focal Person | Per day | 1 | 11 | E |  |  |  |  |  |  |
| District Health Officer | Per day | 1 | 37 | E |  |  |  |  |  |  |
| District Health Inspector | Per day | 1 | 21 | E |  |  |  |  |  |  |
| District Health Visitor | Per day | 1 | 16 | E |  |  |  |  |  |  |
| District Health Educator | Per day | 1 | 18 | E |  |  |  |  |  |  |
| District TB & leprosy supervisor | Per day | 1 | 11 | E |  |  |  |  |  |  |
| District Assistant Drug Inspector | Per day | 1 | 11 | E |  |  |  |  |  |  |
| Senior Nursing Officer | Per day | 1 | 16 | E |  |  |  |  |  |  |
| Medical Superintendent | Per day | 1 | 28 | E |  |  |  |  |  |  |
| Hospital Administrator | Per day | 1 | 16 | E |  |  |  |  |  |  |
| In-charge Adjumani hospital | Per day | 1 | 11 | E |  |  |  |  |  |  |
| In-charge maternity ward | Per day | 1 | 11 | E |  |  |  |  |  |  |
| Faith-based health units | Per day | 1 | 16 | E |  |  |  |  |  |  |
| Secretary for health | Per day | 1 | 11 | E |  |  |  |  |  |  |
| District vector control officer | Per day | 1 | 16 | E |  |  |  |  |  |  |
| Assistant chief administration officer | Per day | 1 | 16 | E |  |  |  |  |  |  |
| Medical officer | Per day | 1 | 11 | E |  |  |  |  |  |  |
| Nursing officer | Per day | 1 | 11 | E |  |  |  |  |  |  |
| Accountant | Per day | 1 | 11 | E |  |  |  |  |  |  |
| Participants transport allowance | Per person | 45 | 6 | F |  |  |  |  |  |  |

^4^ For Adjumani and Jinja, only 50% of the costs were attributed to training, as sensitization activities (shown below) were carried out at the same time.

|  |  | ANC delivery – Adjumani district | | | Campaign delivery – Adjumani district | | | Campaign delivery – Jinja district | | |
| --- | --- | --- | --- | --- | --- | --- | --- | --- | --- | --- |
| Input | Unit | Quantity | Unit Cost (US$)^1^ | Cost Type^2^ | Quantity | Unit Cost (US$)^1^ | Cost Type^2^ | Quantity | Unit Cost (US$)^1^ | Cost Type^2^ |
| TRAINING (continued): | | | | | | | | | | |
| *District training of trainers (continued)* | | | | | | | | | | |
| Participants allowance | Per person | 45 | 11 | F |  |  |  |  |  |  |
| District driver allowance | Per day | 4 | 2 | F |  |  |  |  |  |  |
| District driver salary | Per day | 4 | 7 | E |  |  |  |  |  |  |
| District driver fuel | Per day | 4 | 25 | F |  |  |  |  |  |  |
| National officer per diem | Per day | 6 | 37 | F |  |  |  |  |  |  |
| National facilitators salary | Per day | 6 | 17 | E |  |  |  |  |  |  |
| Malaria Consortium officer per diem | Per day | 6 | 37 | F |  |  |  |  |  |  |
| Malaria Consortium officer salary | Per day | 6 | 55 | F |  |  |  |  |  |  |
| Malaria Consortium driver per diem | Per day | 6 | 37 | F |  |  |  |  |  |  |
| Malaria Consortium driver salary | Per day | 6 | 38 | F |  |  |  |  |  |  |
| Fuel for Malaria Consortium car (return) | Per trip | 1 | 145 | F |  |  |  |  |  |  |
| Car purchase | Per day | 10 | 17 (15) | E |  |  |  |  |  |  |
| Car insurance, tax & maintenance | Per day | 10 | 25 | E |  |  |  |  |  |  |
| Stationary costs | Per person | 50 | 2 | F |  |  |  |  |  |  |
| Hall hire | Hall | 1 | 29 | F |  |  |  |  |  |  |
| Sub-total |  |  | 3,382 (2,527) | E (F) |  |  |  |  |  |  |
| *ANC staff training* | | | | | | | | | | |
| Participants’ salary | Per day | 45 | 141 | E |  |  |  |  |  |  |
| Participants’ food & transport allowance | Per person | 45 | 6 | F |  |  |  |  |  |  |
| Participants’ allowance | Per person | 45 | 6 | F |  |  |  |  |  |  |
| Facilitator per diem | Per day | 12 | 17 | F |  |  |  |  |  |  |
| Facilitators salary | Per day | 12 | 23 | E |  |  |  |  |  |  |
| Car purchase per day | Per day | 12 | 17 (15) | E |  |  |  |  |  |  |
| Car insurance, tax & maintenance | Per day | 12 | 25 | E |  |  |  |  |  |  |
| District driver allowance | Per day | 12 | 2 | F |  |  |  |  |  |  |
| District driver salary | Per day | 12 | 7 | E |  |  |  |  |  |  |
| Fuel | Per day | 12 | 26 | F |  |  |  |  |  |  |
| Stationary | Per person | 270 | 2 | F |  |  |  |  |  |  |
| Hall hire | Per day | 12 | 29 | F |  |  |  |  |  |  |
| Sub-total |  |  | 9,090 (2,062) | E (F) |  |  |  |  |  |  |
| *Sub-county training* | | | | | | | | | | |
| Transport allowance for community medicine distributors | Per person |  |  |  | 348 | 2 | F | 152 | 2 | F |
| Refreshments | Per person |  |  |  | 180 | 3 | F | 79 | 3 | F |
| Per diems trainers | Per day |  |  |  | 6 | 6 | F | 3 | 6 | F |
| Fuel | Per person |  |  |  | 6 | 6 | F | 1.5 | 23 | F |
| Trainers’ salary | Per person |  |  |  | 6 | 14 | E | 3 | 14 | E |
| Motorbike purchase | Per day |  |  |  | 6 | 3 (3) | E (F) | 3 | 3 (3) | E (F) |
| Motorbike insurance & maintenance | Per day |  |  |  | 6 | 2 | E | 3 | 2 | E |
| Sub-total |  |  |  |  |  | 1,248 (1,149) | E (F) |  | 575 (558) | E (F) |

|  |  | ANC delivery – Adjumani district | | | Campaign delivery – Adjumani district | | | Campaign delivery – Jinja district | | |
| --- | --- | --- | --- | --- | --- | --- | --- | --- | --- | --- |
| Input | Unit | Quantity | Unit Cost (US$)^1^ | Cost Type^2^ | Quantity | Unit Cost (US$)^1^ | Cost Type^2^ | Quantity | Unit Cost (US$)^1^ | Cost Type^2^ |
| SENSITIZATION: | | | | | | | | | | |
| *District level* | | | | | | | | | | |
| Per diem for district leaders | Per day |  |  |  | 20 | 6 | F | 20 | 6 | F |
| Central supervisor per diems | Per day |  |  |  | 3 | 37 | F | 1.5 | 37 | F |
| Central supervisors drivers per diem | Per day |  |  |  | 1.5 | 37 | F | 0.5 | 37 | F |
| Central supervisors facilitation allowance | Per day |  |  |  | 2 | 17 | F | 1.5 | 17 | F |
| Venue | Per day |  |  |  | 1 | 14 | F | 0.5 | 14 | F |
| Central supervisors drivers salary | Per day |  |  |  | 1.5 | 18 | E | 0.5 | 18 | E |
| Car purchase cost per day | Per day |  |  |  | 1.5 | 17 (15) | E (F) | 0.5 | 17 (15) | E (F) |
| Car insurance, maintenance & tax | Per day |  |  |  | 1.5 | 25 | E | 0.5 | 25 | E |
| Fuel to district (return) | Per trip |  |  |  | 0.5 | 158 | F | 0.5 | 25 | F |
| Fuel within district | Per day |  |  |  | 1 | 11 | F | 0.5 | 11 | F |
| Sub-total |  |  |  |  |  | 513 (445) | E (F) |  | 271 (249) | E (F) |
| *Sub-county sensitization* | | | | | | | | | | |
| Transport allowance parish leaders | Per person |  |  |  | 40 | 2 | F | 5 | 2 | F |
| Transport allowance Local Council Chairman | Per person |  |  |  | 70 | 2 | F | 38 | 1 | F |
| Refreshments | Per person |  |  |  | 174 | 3 | F | 79 | 3 | F |
| Per diems for trainers | Per day |  |  |  | 6 | 6 | F | 3 | 6 | F |
| Fuel | Per person |  |  |  | 6 | 6 | F | 1.5 | 23 | F |
| Trainers’ salary | Per day |  |  |  | 6 | 14 | E | 3 | 14 | E |
| Motorbike purchase | Per day |  |  |  | 6 | 3 (3) | E (F) | 3 | 3 (3) | E (F) |
| Motorbike insurance & maintenance | Per day |  |  |  | 6 | 2 | E | 3 | 2 | E |
| Sub-total |  |  |  |  |  | 837 (739) | E (F) |  | 387 (338) | E (F) |
| *Other sensitization inputs* | | | | | | | | | | |
| District health officer, facilitation (airtime/fuel) | Per district |  |  |  | 1 | 86 | F | 1 | 86 | F |
| Posters, leaflets and slips | Per LLIN |  |  |  | 16,378 | 0.04 | F | 12,994 | 0.07 | F |
| Sub-total |  |  |  |  |  | 803 | F |  | 1034 | F |

|  |  | ANC delivery – Adjumani district | | | Campaign delivery – Adjumani district | | | Campaign delivery – Jinja district | | |
| --- | --- | --- | --- | --- | --- | --- | --- | --- | --- | --- |
| Input | Unit | Quantity | Unit Cost (US$)^1^ | Cost Type^2^ | Quantity | Unit Cost (US$)^1^ | Cost Type^2^ | Quantity | Unit Cost (US$)^1^ | Cost Type^2^ |
| MANAGEMENT: | | | | | | | | | | |
| *Field office costs* | | | | | | | | | | |
| Programme Coordinator | Per day | 8 | 90 | E |  |  |  |  |  |  |
| Technical Officer | Per day | 10 | 63 | E |  |  |  |  |  |  |
| Technical Officer | Per day | 6 | 55 | E |  |  |  |  |  |  |
| Project Officer | Per day | 8 | 80 | E |  |  |  |  |  |  |
| Project Officer | Per day | 10 | 40 | E |  |  |  |  |  |  |
| Finance/operations officer salary | Per day | 0.6 | 39 | E |  |  |  |  |  |  |
| Driver salary | Per day | 0.6 | 38 | E |  |  |  |  |  |  |
| Finance/operations officer per diem | Per day | 0.6 | 37 | F |  |  |  |  |  |  |
| Driver per diem | Per day | 0.6 | 37 | F |  |  |  |  |  |  |
| Fuel Gulu – Kampala (return) | Per trip | 0.3 | 107 | F |  |  |  |  |  |  |
| Car purchase cost | Per day | 0.6 | 17 (15) | E |  |  |  |  |  |  |
| Car insurance, tax & maintenance | Per day | 0.6 | 25 | E |  |  |  |  |  |  |
| Computer | Per day | 41 | 2 (1) | E (F) |  |  |  |  |  |  |
| Phone and communications | Per day | 41 | 2 | F |  |  |  |  |  |  |
| Bank charges | Per day | 41 | 0.4 | E |  |  |  |  |  |  |
| Rent, electricity, water | Per day | 41 | 1 | E |  |  |  |  |  |  |
| Cleaning/Office supplies | Per day | 41 | 0.7 | E |  |  |  |  |  |  |
| Security | Per day | 41 | 0.5 | E |  |  |  |  |  |  |
| Office stationary | Per day | 41 | 1 | E |  |  |  |  |  |  |
| Sub-total |  |  | 3,126 (233) | E (F) |  |  |  |  |  |  |
| *Kampala office costs* | | | | | | | | | | |
| Prevention Specialist | Per day | 2 | 233 | E | 0.4 | 233 | F | 0.7 | 233 | F |
| Public-Private Partnership Coordinator | Per day | 2 | 152 | E | 2.5 | 152 | F | 4.5 | 152 | F |
| Operations Coordinator | Per day | 1 | 80 | E | 1.4 | 80 | F | 0.7 | 80 | F |
| Senior Operations Officer | Per day |  |  |  | 2.2 | 44 | F | 3.0 | 44 | F |
| Computer | Per day | 5 | 2 (1) | E (F) | 6.5 | 1 | F | 8.9 | 1 | F |
| Phone and communications | Per day | 5 | 11 | E | 6.5 | 11 | F | 8.9 | 11 | F |
| Rent, electricity, water, cleaning, security | Per day | 5 | 8 | E | 6.5 | 8 | E | 8.9 | 8 | E |
| Office stationary and supplies | Per day | 5 | 2 | E | 6.5 | 2 | F | 8.9 | 2 | F |
| Sub-total |  |  | 898 (7) | E (F) |  | 834 (780) | E (F) |  | 1,225 (1,151) | E (F) |

|  |  | ANC delivery – Adjumani district | | | Campaign delivery – Adjumani district | | | Campaign delivery – Jinja district | | |
| --- | --- | --- | --- | --- | --- | --- | --- | --- | --- | --- |
| Input | Unit | Quantity | Unit Cost (US$)^1^ | Cost Type^2^ | Quantity | Unit Cost (US$)^1^ | Cost Type^2^ | Quantity | Unit Cost (US$)^1^ | Cost Type^2^ |
| LLIN purchase, transport, storage, registration and distribution: | | | | | | | | | | |
| LLIN | Per net | 15,188 | 1.9 (5.3) | E (F) | 16,378 | 2.03 (5.75) | E (F) | 12,994 | 1.9 (5.3) | E (F) |
| Truck hire to district | Per net | 1 | 1,614 | F | 1 | 1,740 | F | 1 | 431 | F |
| Truck hire within district | Per net |  |  |  | 1 | 1,218 | F | 1 | 281 | F |
| Storage | Per net |  |  |  | 1 | 113 | E | 1 | 86 | F |
| Sub-total |  |  | 29,857 (81,503) | E (F) |  | 36,318 (97,131) | E (F) |  | 24,967 (69,147) | E (F) |
| *Delivery from district store to health centre* | | | | | | | | | | |
| District officials’ allowance | Per day | 144 | 9 | F |  |  |  |  |  |  |
| District driver per diem | Per day | 72 | 9 | F |  |  |  |  |  |  |
| Fuel | Litres | 1440 | 1 | F |  |  |  |  |  |  |
| Loading LLINs | Per person | 144 | 3 | F |  |  |  |  |  |  |
| Salary malaria focal person | Per day | 72 | 11 | E |  |  |  |  |  |  |
| Salary health educator | Per day | 72 | 18 | E |  |  |  |  |  |  |
| Salary district driver | Per day | 72 | 7 | E |  |  |  |  |  |  |
| Car purchase cost per day | Per day | 72 | 17 (15) | E (F) |  |  |  |  |  |  |
| Car insurance, tax & maintenance per day | Per day | 72 | 25 | E |  |  |  |  |  |  |
| Sub-total |  |  | 9,798 (5,199) | E (F) |  |  |  |  |  |  |
| *Registration* | | | | | | | | | | |
| Community Medicine Distributor allowance | Per day |  |  |  | 7,313 | 2 | F | 152 | 2 | F |
| Sub-county supervisor allowance | Per day |  |  |  | 12 | 6 | F | 6 | 6 | F |
| Sub-county supervisor fuel allowance | Per day |  |  |  | 12 | 6 | F | 6 | 6 | F |
| Sub-county supervisor salary | Per day |  |  |  | 12 | 18 | E | 6 | 18 | E |
| Sub-county supervisor motorbike purchase cost | Per day |  |  |  | 12 | 3 (3) | E (F) | 6 | 3 (3) | E (F) |
| Motorbike insurance & maintenance | Per day |  |  |  | 12 | 2 | E | 6 | 2 | E |
| Central supervisors facilitation allowance | Per day |  |  |  | 8 | 17 | F | 6 | 17 | F |
| Central supervisors per diem | Per day |  |  |  | 8 | 37 | F | 6 | 37 | F |
| Central team’s driver salary | Per day |  |  |  | 6 | 18 | E | 2 | 18 | E |
| Central team’s driver per diem | Per day |  |  |  | 6 | 37 | F | 2 | 37 | F |
| Central supervisor’s car purchase cost | Per day |  |  |  | 6 | 17 (15) | E (F) | 2 | 17 (15) | E (F) |
| Car insurance, tax & maintenance | Per day |  |  |  | 6 | 25 | E | 2 | 25 | E |
| Central supervisor’s car fuel | Per day |  |  |  | 6 | 23 | F | 2 | 23 | F |
| Malaria Consortium supervisor salary | Per day |  |  |  | 6 | 80 | E | 2 | 80 | E |
| Malaria Consortium supervisor per diem | Per day |  |  |  | 2 | 37 | F | 2 | 37 | F |
| Malaria Consortium driver salary | Per day |  |  |  | 2 | 18 | E | 2 | 18 | E |
| Malaria Consortium driver per diem | Per day |  |  |  | 2 | 37 | F | 2 | 37 | F |
| Malaria Consortium car purchase | Per day |  |  |  | 2 | 17 (15) | E (F) | 2 | 17 (15) | E (F) |
| Car insurance, tax & maintenance | Per day |  |  |  | 2 | 25 | E | 2 | 25 | E |
| MC car fuel | Per day |  |  |  | 2 | 23 | F | 2 | 23 | F |
| Sub-total |  |  |  |  |  | 3,127 (2,034) | E (F) |  | 1,508 (1051) | E (F) |

|  |  | ANC delivery – Adjumani district | | | Campaign delivery – Adjumani district | | | Campaign delivery – Jinja district | | |
| --- | --- | --- | --- | --- | --- | --- | --- | --- | --- | --- |
| Input | Unit | Quantity | Unit Cost (US$)^1^ | Cost Type^2^ | Quantity | Unit Cost (US$)^1^ | Cost Type^2^ | Quantity | Unit Cost (US$)^1^ | Cost Type^2^ |
| LLIN purchase, transport, storage, registration and distribution (continued): | | | | | | | | | | |
| *Distribution* | | | | | | | | | | |
| Health facility workers’ time | Per net | 15,188 | 0.06 | E |  |  |  |  |  |  |
| Guard, educator, team leader, allowance | Per day |  |  |  | 7,313 | 3 | F | 80 | 3 | F |
| Community Medicine Distributor allowance | Per day |  |  |  | 318 | 2 | F | 152 | 2 | F |
| Local Council Chairman allowance | Per day |  |  |  | 158 | 2 | F | 76 | 2 | F |
| Sub-county supervisor allowance | Per day |  |  |  | 12 | 6 | F | 4 | 6 | F |
| Sub-county supervisor fuel allowance | Per day |  |  |  | 12 | 6 | F | 4 | 6 | F |
| Sub-county supervisor salary | Per day |  |  |  | 12 | 18 | F | 4 | 18 | E |
| Sub-county supervisor motorbike purchase cost | Per day |  |  |  | 12 | 3 (3) | E | 4 | 3 (3) | E (F) |
| Motorbike insurance & maintenance | Per day |  |  |  | 12 | 2 | E | 4 | 2 | E |
| Central supervisors’ allowance | Per day |  |  |  | 12 | 2 | F | 6 | 17 | F |
| Central supervisors’ per diem | Per day |  |  |  | 12 | 0.5 | F | 6 | 38 | F |
| Central team’s driver salary | Per day |  |  |  | 12 | 18 | E | 2 | 18 | E |
| Central team driver per diem | Per day |  |  |  | 12 | 37 | F | 2 | 37 | F |
| Central supervisor’s car purchase cost | Per day |  |  |  | 6 | 17 (15) | E (F) | 2 | 17 (15) | E (F) |
| Car insurance, tax & maintenance | Per day |  |  |  | 6 | 25 | E | 2 | 25 | E |
| Central supervisor car fuel | Per day |  |  |  | 6 | 23 | F | 2 | 23 | F |
| Malaria Consortium supervisor salary | Per day |  |  |  | 6 | 80 | E | 2 | 80 | E |
| Malaria Consortium supervisor per diem | Per day |  |  |  | 6 | 37 | F | 2 | 37 | F |
| Malaria Consortium driver salary | Per day |  |  |  | 6 | 18 | E | 2 | 18 | E |
| Malaria Consortium driver per diem | Per day |  |  |  | 6 | 37 | F | 2 | 37 | F |
| Malaria Consortium car purchase | Per day |  |  |  | 6 | 17 (15) | E (F) | 2 | 17 (15) | E (F) |
| Car tax, insurance & maintenance | Per day |  |  |  | 6 | 25 | E | 2 | 25 | E |
| MC car fuel | Per day |  |  |  | 6 | 23 | F | 2 | 23 | F |
| Pens | Pen |  |  |  | 159 | 0.1 | F | 76 | 0.1 | F |
| Marker pens & flipcharts | Pen |  |  |  | 3 | 6 | F | 18 | 6 | F |
| Inkpads & ink bottles | Bottle & pad |  |  |  | 102 | 4 | F | 3 | 4 | F |
| String | Roll |  |  |  | 20 | 3 | F |  |  |  |
| Batteries | Set |  |  |  | 120 | 11 | F |  |  |  |
| Printing of manual | Manual |  |  |  | 3 | 0.7 | F | 6 | 0.7 | F |
| Printing stock cards | Card |  |  |  | 3 | 1 | F | 1 | 1 | F |
| Printing registration & distribution forms | Form |  |  |  | 1070 | 0.3 | F | 205 | 0.03 | F |
| Sub-total |  |  | 940 | E |  | 5,935 (4,537) | E (F) |  | 2,015 (1,526) | E (F) |

|  |  | ANC delivery – Adjumani district | | | Campaign delivery – Adjumani district | | | Campaign delivery – Jinja district | | |
| --- | --- | --- | --- | --- | --- | --- | --- | --- | --- | --- |
| Input | Unit | Quantity | Unit Cost (US$)^1^ | Cost Type^2^ | Quantity | Unit Cost (US$)^1^ | Cost Type^2^ | Quantity | Unit Cost (US$)^1^ | Cost Type^2^ |
| LLIN Purchase, transport, storage and distribution (continued): | | | | | | | | | | |
| *Post-distribution follow-up* | | | | | | | | | | |
| Community Medicine Distributor allowance | Per day |  |  |  | 318 | 2 | F | 152 | 2 | F |
| Sub-county supervisors’ per diem | Per day |  |  |  |  |  |  | 4 | 6 | F |
| Sub-county supervisors’ motorbike purchase cost | Per day |  |  |  |  |  |  | 4 | 3 (3) | E (F) |
| Motorbike insurance & maintenance | Per day |  |  |  |  |  |  | 4 | 2 | E |
| Sub-county supervisors’ fuel | Per day |  |  |  |  |  |  | 4 | 6 | F |
| Malaria Focal Persons’ fuel for supervision | Total |  |  |  |  |  |  | 1 | 115 | F |
| Sub-total |  |  |  |  |  | 548 | F |  | 441 (423) | E (F) |
| Overheads | | | | | | | | | | |
| Procurement (5%) |  | 1 | 3,994 | F | 1 | 4,709 | F | 1 | 3,417 | F |
| General (18%) |  | 1 | 2,477 | F | 1 | 2,604 | F | 1 | 1,325 | F |
| Sub-total |  |  | 6,471 | F |  | 7,313 | F |  | 4,742 | F |
