# Supplementary material for: Costs and effects of two public sector delivery channels for long-lasting insecticidal nets in Uganda
Source: Malar J. 2010 Apr 20;9:102. doi: 10.1186/1475-2875-9-102 (PMC2868859; doi:10.1186/1475-2875-9-102)
Supplement: Additional file 2 — Table of costing results. Financial and economic cost per LLIN delivered and cost per TNY. Results are reported both for an assumed 100% retention and use, and adjusted for actual retention and use as observed in the study (see Table 4). Adjusted costs were calculated by reducing the total number of nets delivered by the relevant proportions shown in Table 4 in the rows 'LLINs retained by recipient household' and 'LLINs retained that were slept under the previous night'. [file 1475-2875-9-102-S2.DOC]

**Additional File 2:** Financial and economic cost per LLIN delivered and cost per TNY. Results are reported both for an assumed 100% retention and use, and adjusted for actual retention and use as observed in the study (see Table 5). Adjusted costs were calculated by reducing the total number of nets delivered by the relevant proportions shown in Table 5 in the rows ‘LLINs retained by recipient household’ and ‘LLINs retained that were slept under the previous night.

| **Distribution type** | ANC delivery – Adjumani district | | | | Campaign delivery – Adjumani district | | | | Campaign delivery – Jinja district | | | |
| --- | --- | --- | --- | --- | --- | --- | --- | --- | --- | --- | --- | --- |
| **Number of nets delivered** | 15,188 | | | | 16,378 | | | | 12,994 | | | |
| **Treated net years (TNY)** | 45,564 | | | | 49,134 | | | | 38,982 | | | |
|  | Financial cost (US$) | % total financial cost^1^ | Economic cost (US$) | % total economic cost^1^ | Financial cost (US$) | % total financial cost^1^ | Economic cost (US$) | % total economic cost^1^ | Financial cost (US$) | % total financial cost^1^ | Economic cost (US$) | % total economic cost^1^ |
| **Activity** | | | | | | | | | | | | |
| Training | 0.22 | 20.7 % | 0.51 | 22.5 % | 0.10 | 9.5 % | 0.12 | 9.8 % | 0.06 | 9.1 % | 0.07 | 8.9 % |
| Sensitization & IEC | 0.22 | 20.7 % | 0.51 | 22.5 % | 0.15 | 14.8 % | 0.16 | 12.7 % | 0.16 | 23.5 % | 0.16 | 21.6 % |
| Registration | N/A | N/A | N/A | N/A | 0.12 | 11.9 % | 0.19 | 15.6 % | 0.08 | 12.1 % | 0.12 | 15.3 % |
| Distribution | 0 | 0 % | 0.06 | 2.7 % | 0.28 | 26.6 % | 0.36 | 29.5 % | 0.12 | 17.6 % | 0.16 | 20.4 % |
| Net transport | 0.45 | 42.0 % | 0.75 | 33.1 % | 0.18 | 17.3 % | 0.18 | 14.7 % | 0.05 | 8.2 % | 0.05 | 7.2 % |
| Storage | 0 | 0 % | 0.01 | 0.3 % | 0 | 0 % | 0.01 | 0.6 % | 0.01 | 1.0 % | 0.01 | 0.9 % |
| Project Management | 0.02 | 1.5 % | 0.26 | 11.7 % | 0.05 | 4.6 % | 0.05 | 4.2 % | 0.09 | 13.3 % | 0.09 | 12.4 % |
| Cost of LLIN | 5.26 |  | 1.86 |  | 5.75 |  | 2.03 |  | 5.26 |  | 1.86 |  |
| Procurement Overhead | 0.26 |  | 0.26 |  | 0.29 |  | 0.29 |  | 0.26 |  | 0.26 |  |
| General Overhead | 0.16 | 15.3% | 0.16 | 7.2% | 0.16 | 15.3% | 0.16 | 13.0% | 0.10 | 15.3% | 0.10 | 13.4% |
| Total project cost per LLIN  (i.e. delivery cost + net) | 6.59 |  | 4.39 |  | 7.08 |  | 3.55 |  | 6.19 |  | 2.88 |  |
| Total cost per LLIN delivered  (i.e. excl. cost of net) | 1.07 | 100 % | 2.27 | 100 % | 1.04 | 100 % | 1.23 | 100 % | 0.67 | 100 % | 0.76 | 100 % |
| Cost per TNY |  |  | 1.46 |  |  |  | 1.18 |  |  |  | 0.96 |  |
| Retained LLINs:  Cost per LLIN delivered (excl. net)  Cost per TNY |  |  | 2.29  1.47 |  |  |  | 1.25  1.19 |  |  |  | 0.81  0.98 |  |
| Retained and used LLIN:  Cost per LLIN delivered (excl. net)  Cost per TNY |  |  | 2.31  1.48 |  |  |  | 1.28  1.21 |  |  |  | 1.10  1.11 |  |

^1^ Excluding the cost of the LLIN
