# Supplementary material for: Costs and effects of two public sector delivery channels for long-lasting insecticidal nets in Uganda
Source: Malar J. 2010 Apr 20;9:102. doi: 10.1186/1475-2875-9-102 (PMC2868859; doi:10.1186/1475-2875-9-102)
Supplement: Additional file 3 — Analysis of sensitivity of cost estimates to assumptions. Table showing results of one-way sensitivity analysis [file 1475-2875-9-102-S3.DOC]

**Additional File 3:** Analysis of sensitivity of cost estimates to assumptions

| Assumptions tested | ANC delivery – Adjumani district | | | | Campaign delivery – Adjumani district | | | | Campaign delivery – Jinja district | | | |
| --- | --- | --- | --- | --- | --- | --- | --- | --- | --- | --- | --- | --- |
|  | US$ / LLIN delivered^1^ | % deviation from base case^2^ | US$ / TNY | % deviation from base case^2^ | US$ / LLIN delivered^1^ | % deviation from base case^2^ | US$ / TNY | % deviation from base case^2^ | US$ / LLIN delivered^1^ | % deviation from base case^2^ | US$ / TNY | % deviation from base case^2^ |
| Base case | 4.39 |  | 1.46 |  | 3.55 |  | 1.18 |  | 2.88 |  | 0.96 |  |
| Discount rate |  | | | | | | | | | | | |
| 0 % | 4.28 |  | 1.43 |  | 3.43 |  | 1.14 |  | 2.78 |  | 0.93 |  |
| 10 % | 4.68 | 7 %[[1]](#footnote-2) | 1.56 | 7 % | 3.83 | 8 % | 1.28 | 9 % | 3.14 | 9 % | 1.05 | 9 % |
| Life of LLIN |  | | | | | | | | | | | |
| 2 years | 5.28 | 20 % | 2.64 | 81 % | 4.52 | 27 % | 2.26 | 92 % | 3.77 | 31 % | 1.89 | 97% |
| 5 years | 3.68 |  | 0.74 |  | 2.77 |  | 0.55 |  | 2.17 |  | 0.43 |  |
| Cost of LLIN^3^ |  | | | | | | | | | | | |
| US$ 3.90 | 3.84 | 13 % | 1.28 | 12% | 2.80 | 28 % | 0.93 | 19 % | 2.33 | 19 % | 0.78 | 19% |
| US$ 4.50 | 4.09 |  | 1.36 |  | 3.04 |  | 1.01 |  | 2.58 |  | 0.86 |  |
| US$ 5.50 | 4.49 |  | 1.50 |  | 3.45 |  | 1.15 |  | 2.98 |  | 0.99 |  |

^1^ Economic cost, including the cost of the LLIN

^2^ Only values for greatest % divergence from base case are shown

^3^ The prices shown reflect the cost per LLIN delivered to Kampala, Uganda, depending on the material (polyester or polyethylene) and quantity (large or small volume) procured. These estimates are based on tenders by Malaria Consortium conducted over the last year. US$ 3.90 applies to large volume procurement of polyester LLINs, while US$ 4.50 applies to both large volume polyethylene LLINs or small volume polyester LLINs. US$ 5.50 only applies to small volume polyethylene procurement.

1. [↑](#footnote-ref-2)
